# Impact of diabetes mellitus on osteoarthritis: a scoping review on biomarkers

**DOI:** 10.1017/erm.2024.7

**Published:** 2024-04-12

**Authors:** Shi Rui Seow, Sumaiyah Mat, Amalina Ahmad Azam, Nor Fadilah Rajab, Intan Safinar Ismail, Devinder Kaur Ajit Singh, Suzana Shahar, Maw Pin Tan, Francis Berenbaum

**Affiliations:** 1 Centre for Healthy Ageing and Wellness, Faculty of Health Sciences, Universiti Kebangsaan Malaysia, Kuala Lumpur, Malaysia; 2 Laboratory of Natural Products, Institute of Bioscience, Universiti Putra Malaysia, Serdang, Selangor, Malaysia; 3 ACT4Health Services and Consultancy Sdn. Bhd, Petaling Jaya, Selangor, Malaysia; 4 Division of Geriatric Medicine, Department of Medicine, Faculty of Medicine, University of Malaya, Kuala Lumpur, Malaysia; 5 Rheumatology, Saint-Antoine Hospital, AP-HP, INSERM CSRA, Sorbonne Université, Paris, France

**Keywords:** biomarkers, diabetes mellitus, inflammation, metabolism, osteoarthritis

## Abstract

Osteoarthritis (OA) commonly affects the knee and hip joints and accounts for 19.3% of disability-adjusted life years and years lived with disability worldwide (Refs 1, 2). Early management is important in order to avoid disability uphold quality of life (Ref. 3). However, a lack of awareness of subclinical and early symptomatic stages of OA often hampers early management (Ref. 4). Moreover, late diagnosis of OA among those with severe disease, at a stage when OA management becomes more complicated is common (Refs 5, 6, 7, 8). Established risk factors for the development and progression of OA include increasing age, female, history of trauma and obesity (Ref. 9). Recent studies have also drawn a link between OA and metabolic syndrome, which is characterized by insulin resistance, dyslipidaemia and hypertension (Refs 10, 11).

## Introduction

Osteoarthritis (OA) commonly affects the knee and hip joints and accounts for 19.3% of disability-adjusted life years and years lived with disability worldwide (Refs 1, 2). Early management is important in order to avoid disability uphold quality of life (Ref. 3). However, a lack of awareness of subclinical and early symptomatic stages of OA often hampers early management (Ref. 4). Moreover, late diagnosis of OA among those with severe disease, at a stage when OA management becomes more complicated is common (Refs 5, 6, 7, 8). Established risk factors for the development and progression of OA include increasing age, female, history of trauma and obesity (Ref. 9). Recent studies have also drawn a link between OA and metabolic syndrome, which is characterized by insulin resistance, dyslipidaemia and hypertension (Refs 10, 11).

Diabetes mellitus (DM) is a prevalent non-communicable disease that affects more than 470 million people worldwide (Ref. 12). The presence of diabetes is believed to accelerate the progression of OA and further complicate the management of OA. This has led to the proposal of the ‘diabetes-induced-osteoarthritis’ (DM-OA) phenotype, which suggests that inflammation and oxidative stress predispose persons living with DM to OA (Ref. 13). DM manifests as a chronic hyperglycaemic state which induces further cartilage degeneration and joint inflammation, causing enrichment of advanced glycation end-products (AGEs) and matrix stiffening preventing optimal cushioning of the joint (Ref. 14). This process then contributes to the cycle of worsening of OA symptoms with resultant avoidance of physical inactivity and subsequent weight gain. As a consequence, metabolic dysregulation and joint symptoms persist or worsen (Refs 15, 16).

Biomarker profiles are now one of the tools for quantification of disease activity, for example, procalcitonin for medullary thyroid cancer and tricarboxylic acid from urine metabolites for gastrointestinal diseases (Refs 17, 18). Specifically, an increasing interest has been drawn towards biomarkers for OA with DM (Ref. 19). The identification of novel biomarkers for OA with DM may aid early diagnosis as a key towards improvement in disease outcomes through secondary preventive measures, prior to the onset of irreversible structural changes. Therefore, in this review, we aimed to identify the impact of DM on OA by examining the studied biomarker signatures.

## Methods

### Identification of relevant studies

Literature search was conducted initially in January 2022 and updated in December 2022. Articles containing the key words ‘osteoarthritis’ AND (‘diabetes mellitus’ OR ‘hyperglycemia’) AND ‘biomarker*’ NOT ‘animal model’ were identified from PubMed, Web of Science, EBSCO and the Cochrane library. Complete search syntax is documented in Supplementary Tables S2–S5. Additional full-text articles were identified through cross-referencing of review articles identified through EBSCO. Titles of articles identified were first screened using Rayyan.ai (by three authors independently: SM, AAA and SRS) (Ref. 20). Any disagreement in title screening was resolved through discussion. The abstracts of articles identified from the title search were then screened using Endnote™ (Clarivate, Philadelphia USA, London United Kingdom). The full text of articles for the selected abstracts was subsequently evaluated by two authors (AAA and SRS).

Articles reporting observational studies, including experimental and cross-sectional studies, that investigated any metabolite, intracellular or extracellular matrix component as potential biomarkers in OA with DM were selected. We included studies involving human subjects that utilized samples of synovial fluid, blood, bone and cartilage employing immunoassay, histological and high-performance liquid chromatography techniques.

### Data extraction

AAA and SRS independently extracted data on author, year of publication, study design and population (sample size, gender, inclusion and exclusion criteria, as well as subject grouping), definitions for OA and DM, methodology, specimens collected and signature biomarkers evaluated using a standardized data extraction table. Quality assessment was performed using the modified Newcastle–Ottawa Quality Assessment Scale (Supplementary Tables S6 and S7) (Ref. 21).

## Results

### Study characteristics

**Figure 1.**
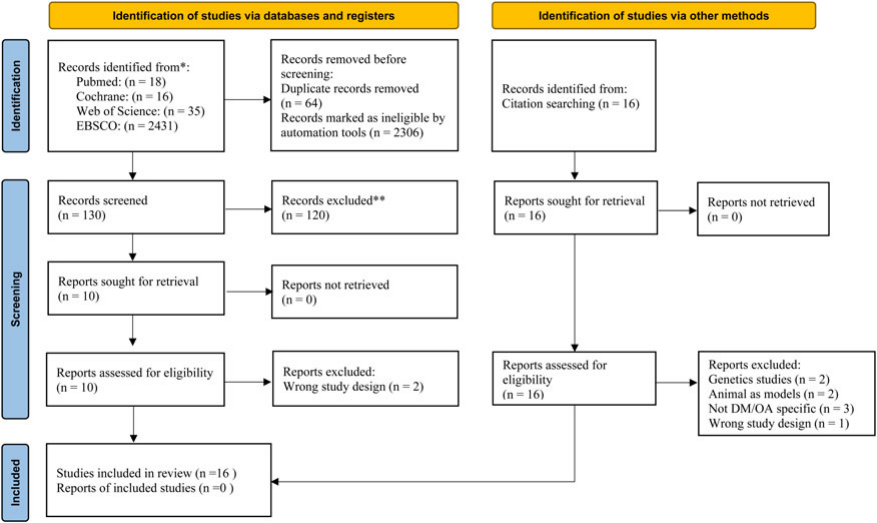
PRISMA flow diagram of study selection.

The database and reference search yielded 16 articles dated until April 2022. Eight articles were first identified from the 2500 potentially eligible articles extracted from PubMed, Web of Science, EBSCO and Cochrane library (Supplementary Figs S1–S4), while another eight articles were identified from cross-referencing. Figure 1 presents the PRISMA flow diagram illustrating the systematic selection of the articles.

The study characteristics were heterogenous across the articles, with the study population sampled from various geographical locations. The total number of participants in each study ranged from 3 to 35 for the experimental studies and 15 to 143 for the cross-sectional studies (Table 1). The studies were conducted in nine countries, six in Europe (Portugal, Spain, Mexico, France, Germany and Finland), two in North America (Canada and the United States) and one in Asia (China). Ten articles identified the presence of DM or hyperglycaemia through medical history, fasting plasma glucose and glycated haemoglobin (HbA1c) levels, whereas DM was not defined by six articles that were based on primary cell cultures.

**Table 1.** Study characteristics

Author, year	Type of study (targeted/untargeted) (cross-sectional/ experimental)	Classification of biomarkers (BIPED)	Quality assessment	Metabolomic approach	Sample (cartilage, bone marrow, synovial tissue/ fluid, blood)	Number of samples
Luo *et al*. (Ref. 22)	Untargeted cross-sectional study **C8A** **CD47** **ANGPTL2** **BSG** **CTSD** **JCAD** **TNC 1018** **TNC 184** **BGN** **FBLN7** **THBS3** **IGHM** **ASPN** **SPARC** **FN1** **COL1A1** **COL6A2**	B	8	LC-MS/MS-based *N*-glycoproteomics analysis	Cartilage	OA + DM+ = 5 OA + DM− = 5 Control = 5
Li *et al*. (Ref. 23)	Targeted cross-sectional study **MMP-13** **ADAMTS5** **GRP78** **ATF6** **TNF-*α*** **IL-6** **NF-*κ*B p65** GLUT-1 **AGEs** **HIF-1*α***	B, P	7	Immunohistochemical staining	Synovial tissue	OA + DM+ = 10 OA + DM− = 10
Scotece *et al*. (Ref. 24)	Targeted cross-sectional study **RBP4** Adipsin Adiponectin Leptin Resistin SF COMP MMP-1 MMP-13 Chitinase-3-like protein 1	D	8	Human ELISA-linked immunosorbent assay	Cartilage	100 OA
Silawal *et al*. (Ref. 25)	Targeted experimental study **Collagen type I** **Collagen type II** **SOX9** **Proteoglycan**	P, D	6	Immunohistochemical staining	hACs	5 OA cultures
Vertti *et al*. (Ref. 26)	Targeted cross-sectional study **SF COMP** **Fasting Plasma Glucose** **HbA1c**	B	10	Human ELISA-linked immunosorbent assay	Synovial fluid	OA + DM+ = 92 OA + DM− = 29
		D			Blood	OA + DM+ = 92 OA + DM− = 29
Eitner *et al*. (Ref. 27)	Targeted cross-sectional study Prostaglandin E2 **IL-6** C-reactive protein HbA1c Plasma triglycerides Uric acid	B, P	9	Human ELISA-linked immunosorbent assay	Synovial fluid	OA + DM+ = 23 OA + DM− = 47
					Serum	OA + DM+ = 23 OA + DM− = 47
Zhang *et al*. (Ref. 28)	Targeted cross-sectional study *N*-(Carboxymethyl)lysine *N*-(Carboxyethyl) lysine **MG-H1** **MG** **Plasma unsaturated phosphatidylcholines, PC ae C34:3 and PC ae C36:3**	B, P	8	Mass spectrometry by flow injection analysis LC–MS/MS	Synovial fluid	OA + DM+ = 46 OA + DM− = 38
		D			Plasma	OA + DM+ = 46 OA + DM− = 38
Hamada *et al*. (Ref. 29)	Targeted experimental study Targeted **TNF** **MMP-1** **MMP-13** **ADAMTS4** **IR** **Akt**	B, P	7	Immunohistochemistry	Synovial tissue	OA + DM+ = 7 OA + DM− = 6
		D		Immunoprecipitation and western blot analysis	Synovial tissue	OA + DM+ = 7 OA + DM− = 6
		D		qRT-PCR	Fibroblast-like-synoviocytes culture	OA + DM− = 4
Ribeiro *et al*. (Ref. 30)	Targeted experimental study **LC3** **p-rpS6** p-Akt IL-1*β* *α*-Tubulin	D	7	Western blotting	Primary human chondrocytes (HC)	OA + DM+ = 4 OA + DM− = 4 Control = 2
Zhang *et al*. (Ref. 31)	Untargeted cross-sectional study **Plasma unsaturated phosphatidylcholines, PC ae C34:3 and PC ae C36:3**	B, P	10	Ultra-high performance liquid-chromatography - mass spectrometry	Synovial fluid	OA + DM+ = 29 OA + DM− = 43 Control = 46
		D			Plasma	OA + DM+ = 29 OA + DM− = 43 DM = 25 Control = 46
Laiguillon *et al*. (Ref. 32)	Targeted experimental study **IL-6** **Prostaglandin E2**	D	9	Human ELISA-linked immunosorbent assay	Homogenous isolated cartilage samples	OA + DM+ = 5 OA + DM− = 5
Tsai *et al*. (Ref. 33)	Targeted experimental study **PKC** Type I TGF receptor **Type II TGF receptor** Smad3 **SOX9** **Aggrecan** **Collagen type II** Collagen type IX	D	6	qRT-PCR and western blot analysis	Human bone marrow-derived MSCs	3 OA
Tsai *et al*. (Ref. 34)	Targeted experimental study **VEGF**	D	7	qRT-PCR and human ELISA-linked immunosorbent assay	Human synovial fibroblast culture	35 OA
Rosa *et al*. (Ref. 35)	Targeted experimental study **MMP-1** **MMP-13** TIMP1 TIMP2 Collagen type I **Collagen type II**		7	qRT-PCR	Cartilage chondrocyte culture	11 OA 7 non-OA
Oren *et al*. (Ref. 36)	Targeted cross-sectional study Leptin Bone alkaline phosphatase Osteocalcin Pyridinium **Cartilage and bone pentosidine** Hydroxylysylpyridinium Lysylpyridinoline HbA1c	B, D, P	10	Human ELISA-linked immunosorbent assay	Serum	OA + DM+ = 10 OA + DM− = 10
					Synovial fluid	OA + DM+ = 10 OA + DM− = 10
				High-performance liquid chromatography	Bone and cartilage	OA + DM+ = 10 OA + DM− = 10
Rosa *et al*. (Ref. 37)	Targeted experimental study **GLUT-1** ROS	D	7	qRT-PCR, western blot and human ELISA-linked immunosorbent assay	Cartilage chondrocyte culture	Non-OA = 15 OA = 18

ADAMTS4, a disintegrin and metalloproteinase with thrombospondin motifs 4; ADAMTS5, a disintegrin and metalloproteinase with thrombospondin motifs 5; AGEs, advanced glycation end-products; Akt, serine threonine kinase; ANGPTL2, angiopoietin-like protein 2; ASPN, asporin; ATF6, activating transcription factor 6; BGN, biglycan; BIPED, burden of disease, investigative, prognostic, efficacy of intervention and diagnostic; BSG, basigin; C8A, C8 alpha chain N437; CD47, cluster of differentiation 47; COL1A1, collagen type 1 alpha 1 chain; COL6A2, collagen type VI alpha 1 chain; CTSD, cathepsin D; FBLN7, ELISA, enzyme-linked immunosorbent assay; fibulin-7; FN1, fibronectin 1; GLUT-1, glucose transporter 1; GRP78, 78 kDa glucose-regulated protein; hAC, human articular chondrocyte; HbA1c, glycated haemoglobin; HIF-1*α*, hypoxia-inducible factor-1*α*; IGHM, immunoglobulin heavy constant mu; IL-1*β*, interleukin-1 beta; IL-6, interleukin-6; IR, insulin receptor; JCAD, junctional cadherin 5-associated protein; LC3, microtubule-associated protein 1A/1B-light chain 3; LC–MS/MS, liquid chromatography coupled-tandem mass spectrometry; MG, methylglyoxal; MG-H1, free methylglyoxal-derived hydroimidazolone; MMP-1, matrix metalloproteinase-1; MMP-13, matrix metalloproteinase-13; MSC, mesenchymal stem cell; NF-*κ*B p65, RelA of nuclear factor kappa-light-chain-enhancer of activated B cells; ROS, reactive oxygen species; p-rpS6, phosphorylated ribosomal S6; qRT-PCR, quantitative real-time polymerase chain reaction; RBP4, retinol binding protein 4; SF COMP, synovial fluid cartilage oligomeric matrix protein; Smad3, SMAD family member 3; SPARC, secreted protein acidic and rich in cysteine/osteonectin; SOX9, SRY-box transcription factor 9; THBS3, thrombospondin 3; TIMP-1, tissue inhibitor of metalloproteinase-1; TIMP-2, tissue inhibitor of metalloproteinase-2; TNC, tenascin C; TNF-*α*, tumour necrosis factor-alpha; VEGF, vascular endothelial growth factor.

Bold biomarkers indicate significant expression change.

The types of samples collected included blood, synovial fluid, bone and cartilage. Five studies used blood samples whereas nine studies collected cartilage or synovial tissues through total knee replacement surgeries and autopsies. The tissues collected were processed into primary cultures to measure in vitro cell expression under hyperglycaemic conditions. The results of this review were categorized according to the following classification: (1) DM-specific biomarkers, (2) cartilage-specific factors, (3) inflammatory mediators, (4) proteases, (5) cell homoeostasis regulators and (6) AGEs and phospholipids.

### Biomarkers screening approach

Three of the 16 selected studies utilized metabolomic analysis and mass spectrometry techniques to screen for candidate markers (Table 1). Mass spectrometry was coupled with the separation techniques of flow-injection and liquid chromatography. Zhang *et al*. evaluated 168/186 biomarkers including 40 acylcarnitines (1 free carnitine), 20 amino acids, 9 biogenic amines, 87 glycerophospholipids, 11 sphingolipids and 1 hexose from plasma and synovial fluid, and eventually proposed plasma unsaturated phosphatidylcholines (PCs), PC ae C34:3 and PC ae C36:3 as possible OA with DM biomarkers after matching the two samples (Ref. 31). As a continuation of the previously untargeted metabolomic approach, AGEs and their precursor were quantified with liquid chromatography coupled-tandem mass spectrometry (LC–MS/MS) in order to identify markers associated with PC ae C34:3 and PC ae C36:3 concentrations (Ref. 28). Luo *et al*. compared the changes in *N*-glycosylated protein abundance from cartilages using LC–MS/MS-based *N*-glycoproteomics analysis and showed 1 upregulated and 16 downregulated *N*-glycosylated peptides between OA and OA with DM groups (Ref. 22).

Oren *et al*. performed high-performance liquid chromatography and enzyme-linked immunosorbent assay (ELISA) for tissue and fluid samples, respectively (Ref. 36). The remaining studies utilized immunological techniques for targeted biomarker detection (Table 1).

Eight studies measured biomarker expression through isolated cell cultures: isolated fibroblast-like synoviocytes and isolated chondrocytes, three of these studies compared results of those with OA only with those with comorbid DM (Refs 23, 29, 30, 32) and five studies isolated samples from individuals with OA only for further high-glucose stimulation culture (Refs 25, 33, 34, 35, 37). Studies by Hamada *et al*. and Tsai *et al*. isolated RNA from treated fibroblast-like synoviocytes, whereas Rosa *et al*. isolated RNA from chondrocyte cultures for quantitative real-time polymerase chain reaction which enabled quantitation of targeted biomarkers (Table 1).

### Method of OA assessment

The articles utilized different criteria to determine the potential presence of OA utilized, including radiographic evidence with or without the Kellgren and Lawrence (KL) grading, the American College of Rheumatology (ACR) clinical criteria or planned total knee arthroplasty. Ten studies recruited participants with OA who were about to have total knee replacement in order to sample synovial fluid, bone or cartilage during their surgery. Six studies did not utilize the ACR clinical diagnostic criteria confirmed by an orthopaedic surgeon and radiographic evidence with KL grading. One of the studies excluded non-OA participants based on weight-bearing anteroposterior and lateral 30° knee flexion radiographic images (Ref. 26).

### Candidate biomarkers

**Figure 2.**
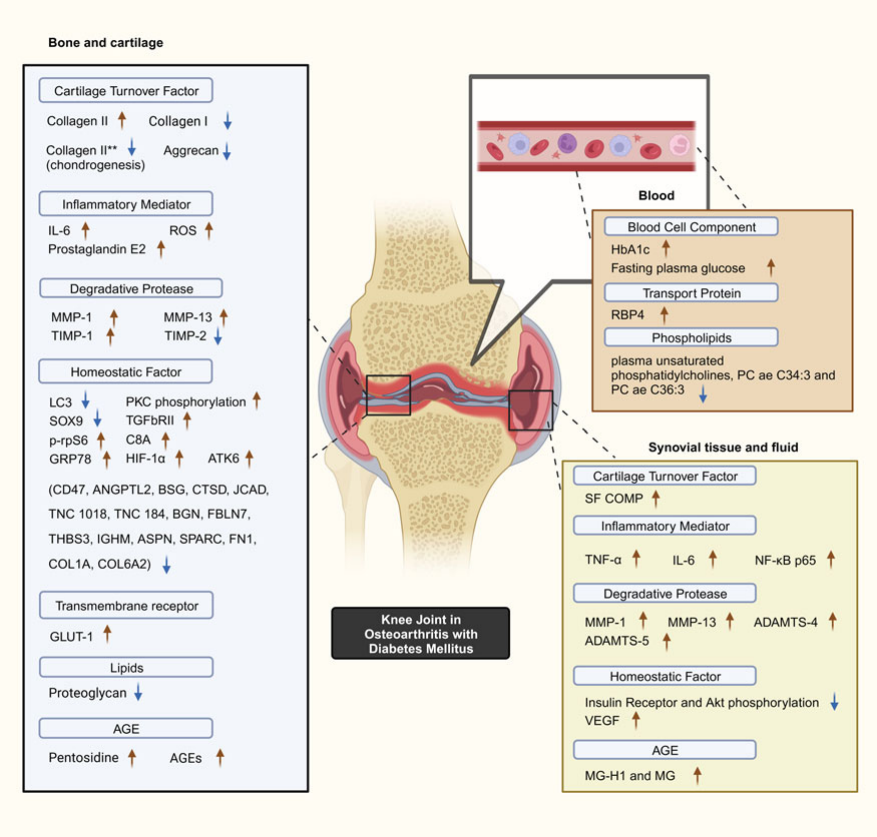
Postulated DM–OA biomarker pathway. An illustrated diagram with the proposed pathway for biomarkers which differentiates the diabetes-OA phenotype from the classical OA phenotype. The biomarkers with significant different in expression magnitude are listed according to sample origin. Red arrows and blue arrows indicate upregulated and downregulated OA with DM biomarker expression as compared with OA, respectively. **Indicates collagen type II expressed by human bone marrow-derived mesenchymal cells during chondrogenesis capacity experiment. Created with BioRender.com. ADAMTS4, a disintegrin and metalloproteinase with thrombospondin motif 4; ADAMTS5, a disintegrin and metalloproteinase with thrombospondin motif 5; AGEs, advanced glycation end-products; Akt, serine threonine kinase; ANGPTL2, angiopoietin-like protein 2; ASPN, asporin; ATF6, activating transcription factor 6; BGN, biglycan; BSG, basigin; C8A, C8 alpha chain N437; CD47, cluster of differentiation 47; COL1A1, collagen type 1 alpha 1 chain; COL6A2, collagen type VI alpha 1 chain; CTSD, cathepsin D; FBLN7, fibulin-7; FN1, fibronectin 1; GLUT-1, glucose transporter; GRP78, 78 kDa glucose-regulated protein; HbA1c, glycated haemoglobin; HIF-1*α*, hypoxia-inducible factor-1*α*; IGHM, immunoglobulin heavy constant mu; IL-6, interleukin-6; LC3, microtubule-associated protein 1A/1B-light chain 3; JCAD, junctional cadherin 5-associated protein; MG, methylglyoxal; MG-H1, free methylglyoxal-derived hydroimidazolone; MMP-1, matrix metalloproteinase-1; MMP-13, matrix metalloproteinase-13; NF-*κ*B p65, RelA of nuclear factor kappa-light-chain-enhancer of activated B cells; PKC, protein kinase C; p-rpS6, phosphorylated ribosomal S6; RBP4, retinol binding protein 4; ROS, reactive oxygen species; SF COMP, synovial fluid cartilage oligomeric matrix protein; Smad3, SMAD family member 3; SPARC, secreted protein acidic and rich in cysteine/osteonectin; SOX9, SRY-box transcription factor 9; THBS3, thrombospondin 3; TIMP-1, tissue inhibitor of metalloproteinase-1; TIMP-2, tissue inhibitor of metalloproteinase-2; TNC, tenascin C; TNF-*α*, tumour necrosis factor-alpha; TGF*β*RII, type II transforming growth factor-*β* receptor; VEGF, vascular endothelial growth factor.

Selected studies quantified key biomarker expression in terms of the presence of significant increases or decreases corresponding to the presence of DM with OA as a constant. Minimal overlap existed between the studies with regards to biomarkers studied. Common OA and inflammation biomarkers are observed to be significantly influenced by the presence of DM (Fig. 2).

#### DM-specific biomarkers

As presumed, HbA1c was higher in the group with both OA and diabetes as compared with the group without DM (Ref. 36). Remarkably, population having both DM and OA has significantly higher HbA1c than population without OA (Ref. 26). Next, glucose transporter 1 (GLUT-1) expression reduction in response to high-glucose cultivation was reported in normal chondrocytes but was not observed in OA chondrocytes (Refs 23, 37). Blunted insulin receptor (IR) and serine threonine kinase (Akt) phosphorylation was also observed in synovial cells in response to high-insulin levels, leading to significantly decreased human articular chondrocyte (hAC) proliferation (Refs 25, 29). Retinol binding protein 4 (RBP4) adipokine expression was detected within blood, synovial fluid and cartilage samples of 100 individuals with OA, which was associated with adiponectin, leptin, resistin, matrix metalloproteinase-1 (MMP-1), matrix metalloproteinase-3 (MMP-3), chitinase 3-like-1 and adipsin (Ref. 24).

#### Cartilage-specific factors

Synovial fluid cartilage oligomeric matrix protein (SF COMP) levels were significantly higher in 92 OA with DM subjects compared with 29 OA subjects. At baseline, Rosa *et al*. reported a 3.5-fold higher ratio of collagen type II to type I messenger-ribonucleic acid (mRNAs) expressions in normal chondrocytes as compared with OA origins. Next, transient increase of collagen type II mRNA levels was significant at 24 h in both normal and OA chondrocytes cultured in elevated glucose concentration, but soon reduced to level equivalent to regular glucose concentration cultures. Collagen II production was increased in OA chondrocytes when proceeded with transforming growth factor-*β* (TGF-*β*) stimulation (Ref. 35). On the other hand, Silawal *et al*. observed significantly lower collagen type II expression in hyperglycaemic hAC culture in response to interleukin-10 (IL-10) and high insulin when compared with normal glycaemic culture. Also, compared with normal glycaemic hAC culture, the decrease induced expression of non-specific dedifferentiation marker collagen type I in hyperglycaemic hAC culture decreased more to a significant level after treated with IL-10. Proteoglycan expression decreased in hAC cultured in IL-10-treated media regardless of glycaemic condition (Ref. 25). Chondrogenic capacity analysis of human bone marrow-derived mesenchymal stem cells (MSCs) reported significantly lower aggrecan mRNA expression at day 9 in high-glucose-maintained culture compared with low-glucose-maintained culture. At day 22, both aggrecan and collagen type II mRNA expression, but not collagen type IX, attained statistical significance lower expression in high-glucose-maintained culture (Ref. 33).

#### Inflammatory mediators

Tumour necrosis factor-*α* (TNF-*α*), interleukin-6 (IL-6) and nuclear factor kappa-light-chain-enhancer of activated B cells (NF-*κ*B) in synovial tissue have been found to be higher in individuals with OA and DM compared with individuals with OA (Ref. 23). This is consistent with the immunohistochemistry findings that observed higher TNF expression in fibroblast-like synoviocytes of comparable groups (Ref. 29). Greater IL-6 and prostaglandin E2 expression was also observed in interleukin-1*β* (IL-1*β*)-stimulated human OA with DM cartilage culture compared with OA cartilage culture (Ref. 32). Further, increased IL-6 expression is positively associated with pain in OA with DM (Ref. 27).

Reactive oxygen species (ROS) increased remarkably when cultured cartilage chondrocytes from individuals with OA were stimulated with IL-1*β* to promote glucose transportation, which is said to mimic defective GLUT-1 downregulation and intracellular accumulation of glucose. ROS production in OA chondrocytes was increased and more sustained in glucose-rich culture (Ref. 37).

#### Protease

MMP-13 and a disintegrin and metalloproteinase with thrombospondin motif 5 (ADAMTS5) were significantly upregulated in OA with DM synovial tissue compared with non-DM OA participants (Ref. 23). Similarly, primary OA fibroblast-like synoviocytes treated with TNF has shown increased MMP-1, MMP-13 and a disintegrin and metalloproteinase with thrombospondin motif 4 (ADAMTS4) expression under glucose-rich conditions. Nevertheless, the increased expressions reduced >50% after treating with insulin (Ref. 29). Exposure to glucose-rich conditions increased tissue inhibitor of metalloproteinase-2 (TIMP-2), MMP-1 and MMP-13 (mRNAs in OA chondrocytes), whereas only MMP-1 increment and tissue inhibitor of metalloproteinase-1 (TIMP-1) decrement was observed in normal chondrocytes (Ref. 35).

#### Cell homoeostasis regulators

Within the DM-OA phenotype, chondrocyte expression of microtubule-associated protein 1A/1B-light chain 3 (LC3) was significantly reduced whereas phosphorylated ribosomal S6 (p-rpS6) expressions significantly increased in comparison with both healthy as well as OA chondrocytes (Ref. 30). A reduction in chondrogenic expression was observed in human bone marrow-derived MSCs within high-glucose culture, with an increased protein kinase C (PKC) activation and type II TGF-*β* receptor (TGF*β*RII) expression (Ref. 33). High glucose also induces vascular endothelial growth factor (VEGF) production in human OA synovial fibroblast (Ref. 34). As endoplasmic reticulum stress-related proteins, both activating transcription factor 6 (ATF6) and 78 kDa glucose-regulated protein (GRP78) expression were found to be significantly higher in the OA group with DM as compared with the non-DM group (Ref. 23). The same research population also had higher hypoxia-inducible factor-1*α* (HIF-1*α*) in the OA with DM group, suggesting possible shared pathophysiology of OA with DM (Ref. 23).

hAC and chondrosarcoma cell lines used in IL-10-stimulated cell culture demonstrated reduced proliferation ability in hyperglycaemia and hyperinsulinaemia compared with normal glycaemic conditions. SRY-box transcription factor 9 (SOX9) synthesis under hyperglycaemic conditions was significantly reduced alongside proteoglycan (Ref. 25). Based on a chondrogenesis stimulation using high-glucose-maintained human bone marrow-derived MSCs, SOX9 mRNA expression that reflect chondrogenic capacity was significantly downregulated at day 9 as compared with low-glucose maintained culture, but the significance waned at day 22 (Ref. 33). Among the 17 *N*-glycosylated proteins that shown fold changes between OA and OA with DM group comparison, fibronectin 1 (FN1), collagen type I alpha 1 chain (COL1A1) and collagen type VI alpha 1 chain (COL6A2) played the most central role in the protein–protein interactions; the other significant *N*-glycosylated proteins that likely to participate in OA with DM pathogenesis is C8 alpha chain (C8*α*) N437 (Ref. 22).

#### AGEs and phospholipids

Three AGEs were identified as potential OA with DM biomarkers: free methylglyoxal-derived hydroimidazolone-1 (MG-H1), methylglyoxal (MG) and pentosidine. Zhang *et al*. observed a negative relationship between plasma in participants with both OA and DM and two unsaturated PCs, PC ae C34:3 and PC ae C36:3 (Ref. 31), and subsequently uncovered a positive upregulation of MG-H1 and MG in synovial fluid of participants with OA and DM (Ref. 28). Pentosidine was significantly higher in both the bone and cartilage of OA with DM individuals compared with non-DM OA individuals (Ref. 36). In addition, following high-GLUT-1 expression in Li *et al*.'s study, significantly higher accumulation of AGEs in subjects with OA and DM was found (Ref. 23).

### Burden of disease, investigative, prognostic, efficacy of intervention and diagnostic classification

The burden of disease, investigative, prognostic, efficacy of intervention and diagnostic (BIPED) biomarker system has been widely used to classify OA biomarkers (Ref. 38). Table 1 lists the function of candidate biomarkers identified within the published articles using the BIPED classification. Burden of disease biomarkers may be useful for early detection whereas transcription factors and protein kinase involved in cartilage homoeostasis are considered indicators of OA with DM prognosis. No specific biomarker has been proposed as investigative and efficacy of intervention biomarkers as the selected criteria had not taken into account research on the effects of pharmacological agents.

### Quality assessment

The Newcastle–Ottawa Quality Assessment Scale was used (Ref. 21), and to address heterogeneity, this was modified into separate versions for experimental and cross-sectional studies. The eight items within the scale assess the three domains: participant sampling, comparability and outcomes. Three studies (20%) were assigned the maximal score of 10, whereas two studies (13%) had a score of nine. Seven studies (46%) did not adequately address comparability for confounding factors and hyperglycaemic conditions whereas two studies (13%) controlled for basic confounding factors only (Table 1).

## Discussion

This review has provided a comprehensive catalogue of investigated blood, synovial fluid, bone and cartilage biomarkers for OA with DM. Biomarkers were uniquely evaluated from the perspective of their physiological functions and general structures, in comparison with previous reviews which addressed signature biomarkers in OA; this review article has focused primarily on biomarkers involved in the contribution of DM to joint inflammation and degeneration within OA (Ref. 39). The presence of DM significantly alters biomarker expression in individuals with OA which can be distinguished from basic OA phenotype, the differences may in turn help unravel the mechanisms underlying the acceleration in OA development associated with DM (Ref. 11).

Although cartilage- and synovial-specific factors are present in both classical OA and OA with DM, the two phenotypes are differentiated by the magnitude of biomarker expression; for instance, higher SF COMP levels in OA with DM indicates greater articular cartilage degradation (Refs 40, 41). In contrast, synthesis of proteoglycan in OA hAC was lowered in hyperglycaemia with IL-10. The transient increase of collagen type II mRNA in high-glucose concentration could be rationalized by the depletion of glucose overtime, where mRNA expression at 72 h became similar to that in regular glucose concentration cultures (Ref. 25). Furthermore, the measurement of mRNA might not reflect collagen type II protein concentration (Ref. 42). Expression of collagen type II in OA culture significantly increased when treated with TGF and high-glucose level showing how glucose concentration affect chondrocyte anabolic and catabolic gene expression (Ref. 35). The expression, however, reduced significantly when cultured with high insulin and IL-10 even though IL-10 is known for its chondroprotective effects (Ref. 43), suggesting hyperglycaemia and hyperinsulinaemia impaired chondrocyte expression (Ref. 44). This suppressive capacity also being suggested for the diminished collagen type I observed in OA hAC culture after IL-10 and high-insulin treatment, which usually expressed in monolayer chondrocyte culture and indicates cartilage differentiation activity (Ref. 45). A paradoxical increase in TGF-stimulated collagen type II expression, conversely, is observed within high-glucose culture environments (Ref. 35). Chondrogenic capacity measurement of lower aggrecan and collagen type II mRNA expression in high-glucose-maintained human mesenchymal cells, which are also the two main components of articular extracellular matrix, suggested remarkable influence of high-glucose concentration on chondrogenesis and cartilaginous matrix production, leading to disrupted cartilage homoeostasis as in OA (Ref. 46).

Transcriptional factors and protein kinase expression involved in the chondrocyte life cycle are altered in OA with DM. Decreased LC3 and increased p-rpS6 expression are seen in the chondrocytes of individuals with both OA and DM, which has been attributed to defective autophagy (Refs 30, 47). In the absence of effective autophagy, dysfunctional organelles and macromolecules cannot be removed, which indicates a negative disease prognosis (Refs 48, 49). Higher PKC phosphorylation is observed with high-glucose-maintained MSCs prior to chondrogenesis (Ref. 33). PKC-mediated mitogen-activated protein kinase, activated by TGF-*β*-stimulated Wnt-5a overexpression, signals for chondrogenic differentiation into functional cells (Ref. 50). VEGF is hypothesized to mediate cartilage catabolism and endochondral ossification in OA (Ref. 51), and since VEGF upregulation is significant under hyperglycaemic conditions (Ref. 52), the higher VEGF expression in OA with DM compared with OA is coherent (Ref. 34). SOX9, a chondrocyte-protecting factor commonly downregulated in OA, is further reduced in OA with DM (Refs 25, 53).

The mechanism of endoplasmic reticulum stress to DM is hypothesized that nutrient stress and inflammatory cytokines induce unfolded protein response and endoplasmic reticulum stress by *β*-cell islets of Langerhans, which then stimulate inflammatory response (Ref. 54). The higher expression of ATF6 and GRP78 in the OA with DM group compared with the non-DM group was in line with their endoplasmic reticulum stress regulation roles (Ref. 55). The fact that endoplasmic reticulum stress is involved in OA pathological changes may explain the expression magnitude in OA with DM (Ref. 56).

Inflammation is now considered the key pathway for OA with DM (Ref. 57). The biomarkers identified in the published studies have, however, primarily been associated with insulin resistance and nutrient stress with only a few biomarkers linked with inflammation. Inflammatory mediators shown in Figure 2 have been identified as biomarkers for OA with DM. The chronic hyperglycaemic condition of DM is postulated to be associated with increased expression of inflammatory mediators which has been purported to arise from the interaction between AGEs and macrophages (Ref. 58). Receptor binding for advanced glycation end-products activates pro-inflammatory M1 macrophages to increase NF-*κ*B transcriptional factor, which in turns further enhances TNF-*α* and IL-1*β* expression (Refs 59, 60, 61). Macrophage activation induces TIMPs, MMPs and ADAMTS production, which is responsible for the catabolic action on cartilage, the degradation subsequently releases damage-associated molecular pattern that stimulates inflammatory mediators' production in return (Refs 62, 63, 64, 65).

The novel biomarkers for early OA with DM detection are putatively DM-specific biomarkers. Blunted insulin-dependent phosphorylation of IRs and Akt were observed in the presence of DM, reflecting insulin resistance (Ref. 29). Under normal conditions, IRs undergo trans-autophosphorylation triggered through IR binding, activating the PI3K–PKB/Akt signalling cascade. On the contrary, blunted IR responsiveness and Akt phosphorylation result in defective glucose uptake (Ref. 66). Excessive nutrient stress hyperactivates the mammalian target of rapamycin complex 1, leading to a negative feedback loop that inhibits Akt (Ref. 67). Subsequently, a shift in anti-inflammatory M2-polarized macrophages towards M1-polarization, which is pro-inflammatory, is then observed (Refs 58, 68).

The common diabetes biomarkers glucose and HbA1c levels represent indicators of nutrient stress in OA with DM (Ref. 26). Insulin resistance stimulate ROS production by reducing AMP-activated protein kinase activity in macrophages, which then activates more HIF-1*α* and upregulates glycolysis in the attempt to restore cellular energy homoeostasis, with the eventual increase in glucose-6-phosphate and nicotinamide adenine dinucleotide phosphate production, further promoting ROS production (Refs 69, 70). Within a high-glucose environment, OA chondrocytes fails to downregulate GLUT-1, resulting in intracellular glucose accumulation and ROS production (Ref. 37). RBP4 that contributes to insulin resistance development could be associated with OA development through MMP expression signalling (Refs 71, 72, 73). Plasma unsaturated phosphatidylcholine depletion, linked to increased insulin resistance and reduced cartilage lubrication, has emerged as a potential indicator owing to its greater depletion in OA with DM (Refs 74, 75).

Next, biomarkers resulting from *N*-glycosylation post-translational modification (Ref. 76) have demonstrated significant fold changes in OA with DM cartilage (Ref. 22). FN1 is an extracellular matrix component with vital functions in regulating cell signalling, growth and differentiation (Ref. 77). Distinctly, upregulated FN1 *N*-glycosylation commonly reported in OA is observed to be downregulated in OA with DM (Refs 22, 78). Three *N*-glycosylated PI3K/Akt pathway proteins: FN1, COL1A1 and COL6A2 are downregulated in OA with DM (Refs 22, 79, 80), indicating pathological roles of PI3K/Akt signalling and collagen glycosylation may be different in OA and OA with DM (Ref. 81). Only C8*α* N437 is upregulated in OA with DM (Ref. 22). Notably, C8*α* involves in complement activation and complement complex formation, which is a key modulator in metabolic diseases (Ref. 82).

### Limitation

The non-inclusive of articles not published in the English language limits the scope of this article in recommending putative OA with DM biomarkers. Next, the temporal relationship between OA and DM was not clarified during participant recruitments, and there were potential confusions between markers about their specificities towards OA or DM, raising doubt in the reflecting direction of the biomarkers. Moreover, identified signatures of the specimen harvested from surgical-removed cartilage and stimulation experiments with isolated chondrocytes are not complementary to an early preventive strategy. Future studies should therefore focus on populations with early-stage OA to identify putative diagnostic biomarkers for early OA with DM.

## Conclusion

The novel biomarkers proposed in the scoping review comprise DM-specific biomarkers and cartilage cell homoeostasis regulators with expressions significantly altered in OA with DM as compared with OA. Future studies are required to evaluate the pathophysiology underlying OA with DM by examining the interaction of these biomarkers.

## Supporting information

Seow et al. supplementary material Seow et al. supplementary material

## Supplementary material

For supplementary material accompanying this paper visit http://doi.org/10.1017/erm.2024.7. click here to view supplementary material
