# Supplementary material for: Impact of diabetes mellitus on osteoarthritis: a scoping review on biomarkers
Source: Expert Rev Mol Med. 2024 Apr 12;26:e8. doi: 10.1017/erm.2024.7 (PMC11062141; doi:10.1017/erm.2024.7)
Supplement: Seow et al. supplementary material [file S1462399424000073sup001.docx]

Table S 1 Topic-specific search terms

| Diabetes Mellitus | |
| --- | --- |
| 1 | Hyperglycemia |
| 2 | Hyperglycaemia |
| Osteoarthritis | |
| 3 | Knee Osteoarthritis |
| Biological matrix | |
| 4 | blood |
| 5 | serum |
| 6 | urine |
| 7 | plasma |
| 8 | Synovial fluid |
| 9 | Cartilage |
| 10 | Bone |
| Analytical technique | |
| 11 | spectr* |
| 12 | chromatography |
| 13 | Mass spectrometry |
| 14 | Enzyme-linked immunosorbent assay (ELISA) |
| 15 | nmr |
| 16 | nuclear magnetic resonance |
| 17 | magnetic resonance |
| 18 | Western Blot |
| 19 | Immunohistochemistry staining |

Table S 2 PubMed syntax

| Set | Concept | Search Statement |
| --- | --- | --- |
| 1 | Osteoarthritis | Osteoarthritis[mh] |
| 2 | Diabetes Mellitus | Diabetes Mellitus[mh] OR Hyperglycaemia[mh] |
| 3 | Biomarker | Biomarker*[mh] |
| 4 | Exclusion criteria | model, animal[mh] |
| 5 | Combine sets | (1 AND 2) AND 3 NOT 4 |
|  |  |  |
| 10 | Query | ((Osteoarthritis[MeSH Terms]) AND ((Diabetes Mellitus[MeSH Terms]) OR (Hyperglycaemia[MeSH Terms]))) AND (Biomarker*[MeSH Terms]) NOT (model, animal[MeSH Terms]) |

* = truncation character (wildcard)

mh = MeSH terms

Table S 3 *Cochrane* Syntax

| Set | Concept | Search Statement |
| --- | --- | --- |
| 1 | Osteoarthritis | Osteoarthritis[ti,ab,kw] |
| 2 | Diabetes Mellitus | Diabetes Mellitus[ti,ab,kw] OR Hyperglycaemia[ti,ab,kw] |
| 3 | Biomarker | Biomarker*[ ti,ab,kw] |
| 4 | Exclusion criteria | Animal models[ti,ab,kw] |
| 5 | Combine sets | (1 AND 2) AND 3 NOT 4 |
|  |  |  |
| 6 | Query | Osteoarthritis[ ti,ab,kw] AND (Diabetes Mellitus[ ti,ab,kw] OR Hyperglycaemia[ ti,ab,kw]) AND Biomarker*[ ti,ab,kw] NOT animal models[ ti,ab,kw] |

 * = truncation character (wildcard)

ti,ab,kw = limit to title, abstract fields and keywords (author keywords and keywords Plus^®^)

Table S 4 Web of Science Syntax

| Set | Concept | Search Statement |
| --- | --- | --- |
| 1 | Osteoarthritis | TS=(Osteoarthritis) |
| 2 | Diabetes Mellitus | TS=(Diabetes Mellitus) OR TS=(Hyperglycaemia) |
| 3 | Biomarker | TS=(Biomarker*) |
| 4 | Exclusion criteria | TS=(Model, animal) |
| 5 | Inclusion criteria | Humans(MeSH headings) AND English(Languages) |
| 7 | Combine sets | (((1 AND 2) AND 3) NOT 4) AND 5 |
|  |  |  |
| 8 | Query | {[(TS=(Osteoarthritis) AND (TS=(Diabetes Mellitus) OR TS=(Hyperglycaemia))) AND TS=(Biomarker*)] NOT TS=(Models, animal)} AND English(Languages) |

 * = truncation character (wildcard)

TS = Topic

Table S 5 *EBSCO* syntax (MEDLINE Ultimate)

| Set | Concept | Search Statement |
| --- | --- | --- |
| 1 | Osteoarthritis | Osteoarthritis |
| 2 | Diabetes Mellitus | Diabetes Mellitus OR Hyperglycaemia |
| 3 | Biomarker | Biomarker* |
| 4 | Exclusion criteria | Animal models |
| 5 | Inclusion criteria | Humans AND English AND Academic journals AND Magazines AND Books |
| 7 | Combine sets | (((1 AND 2) AND 3) NOT 4) AND 5 |
|  |  |  |
| 9 | Query | Osteoarthritis AND (Diabetes Mellitus OR Hyperglycaemia) AND Biomarker* NOT Animal models AND Humans AND English AND Academic journals |

* = truncation character (wildcard)

Figure S 1 PubMed syntax


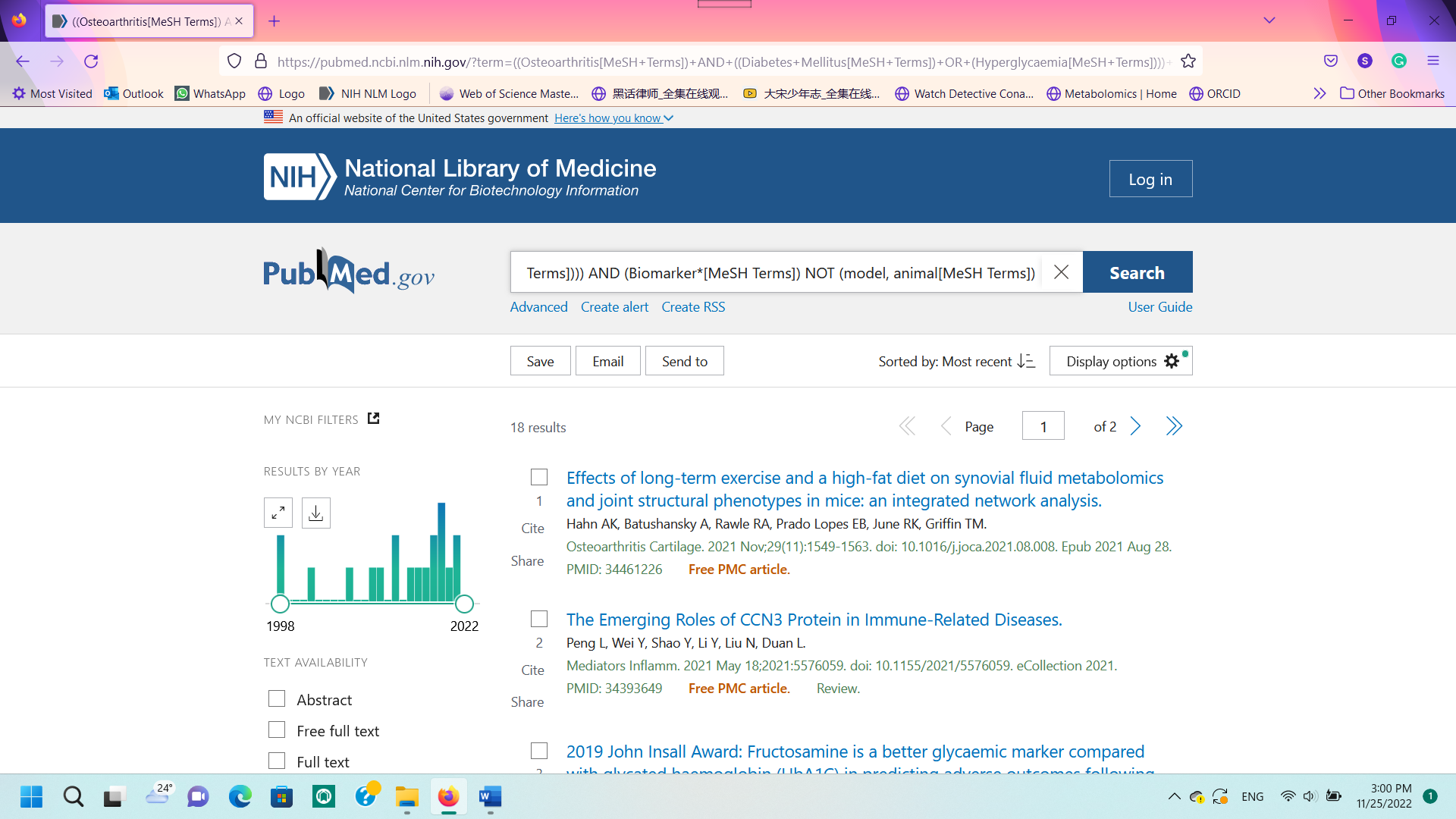


Figure S 2 *Cochrane* Syntax


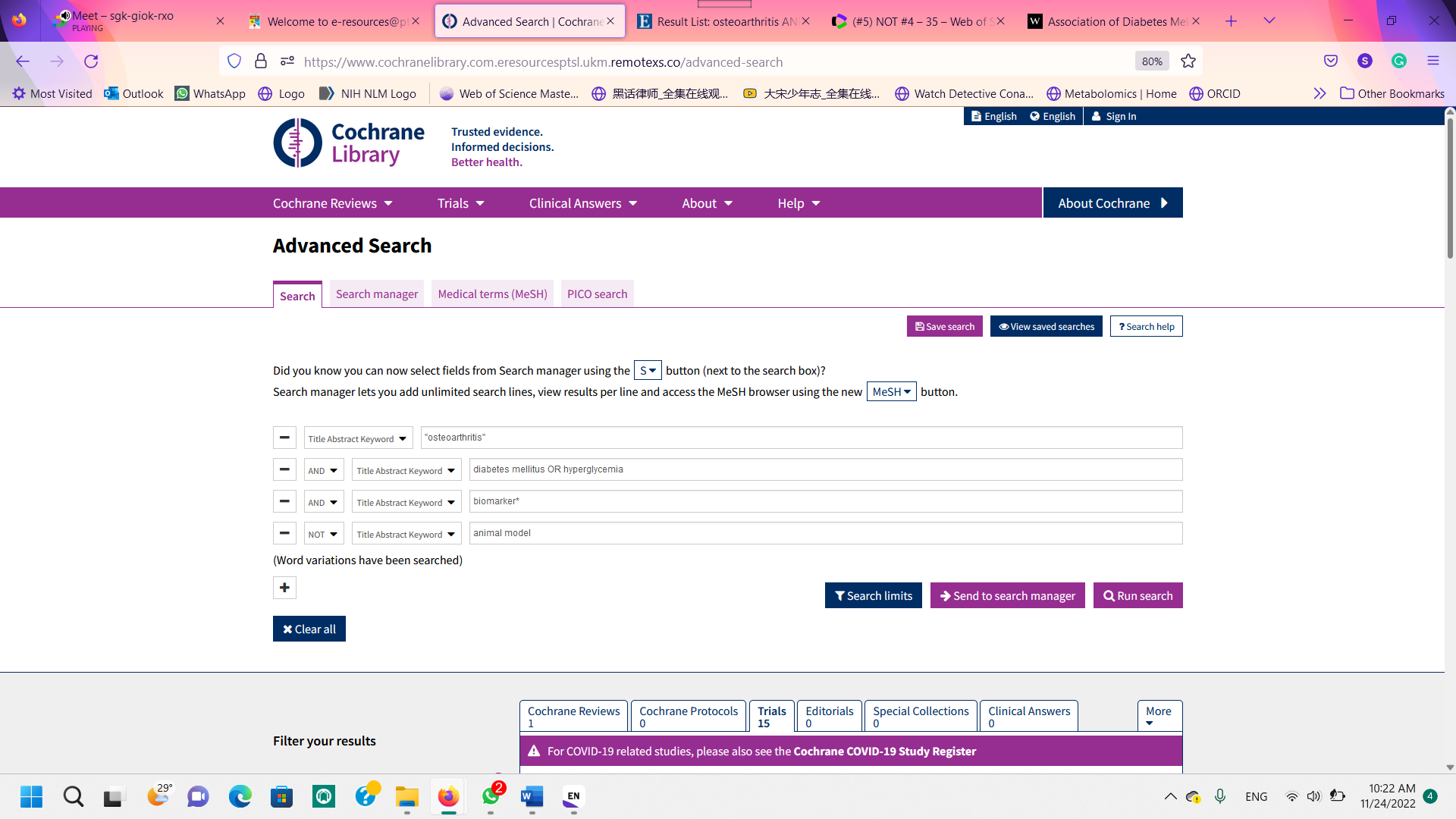


Figure S 3 Web of Science Syntax


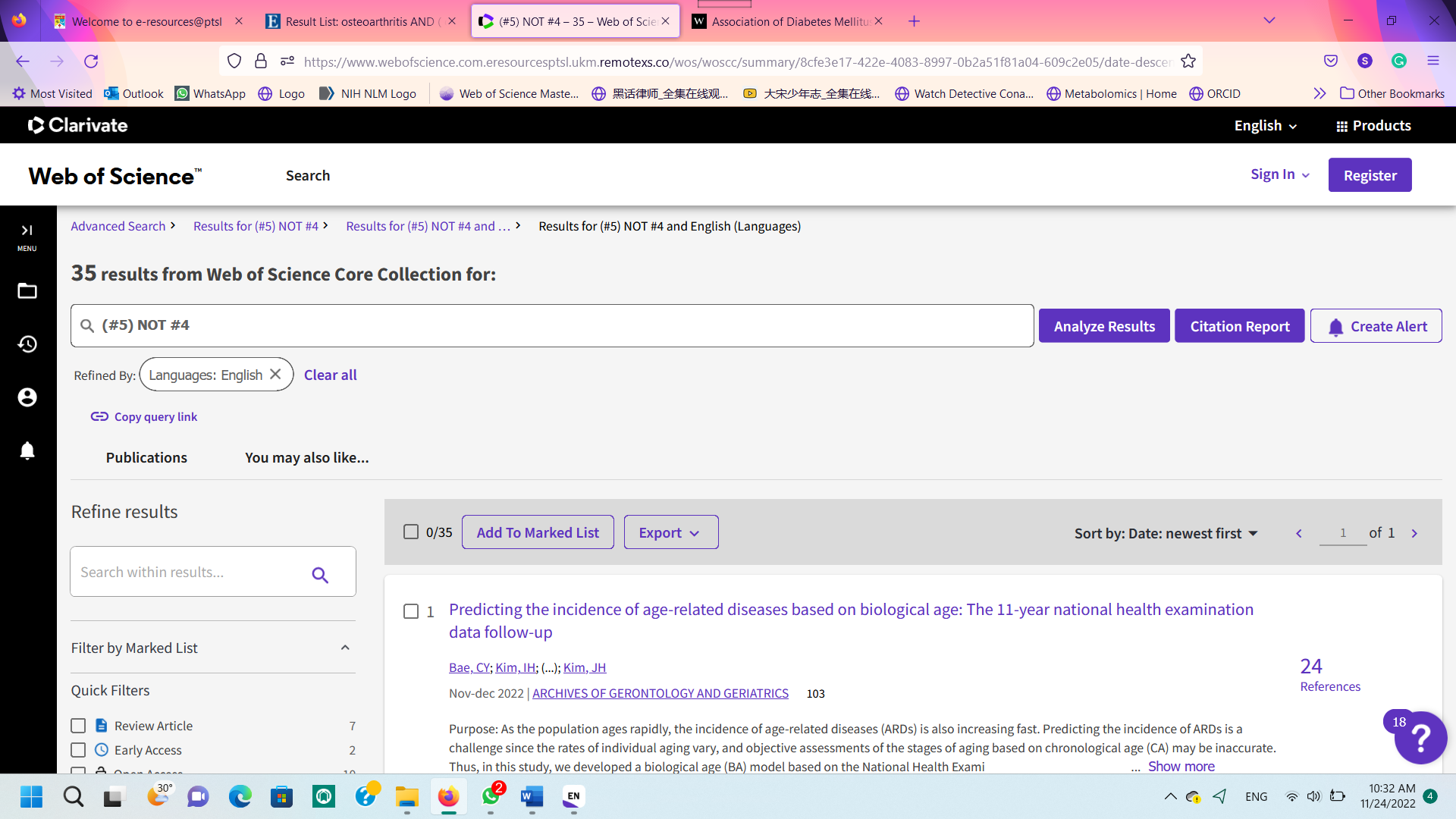


Figure S 4 *EBSCO* syntax (MEDLINE Ultimate)


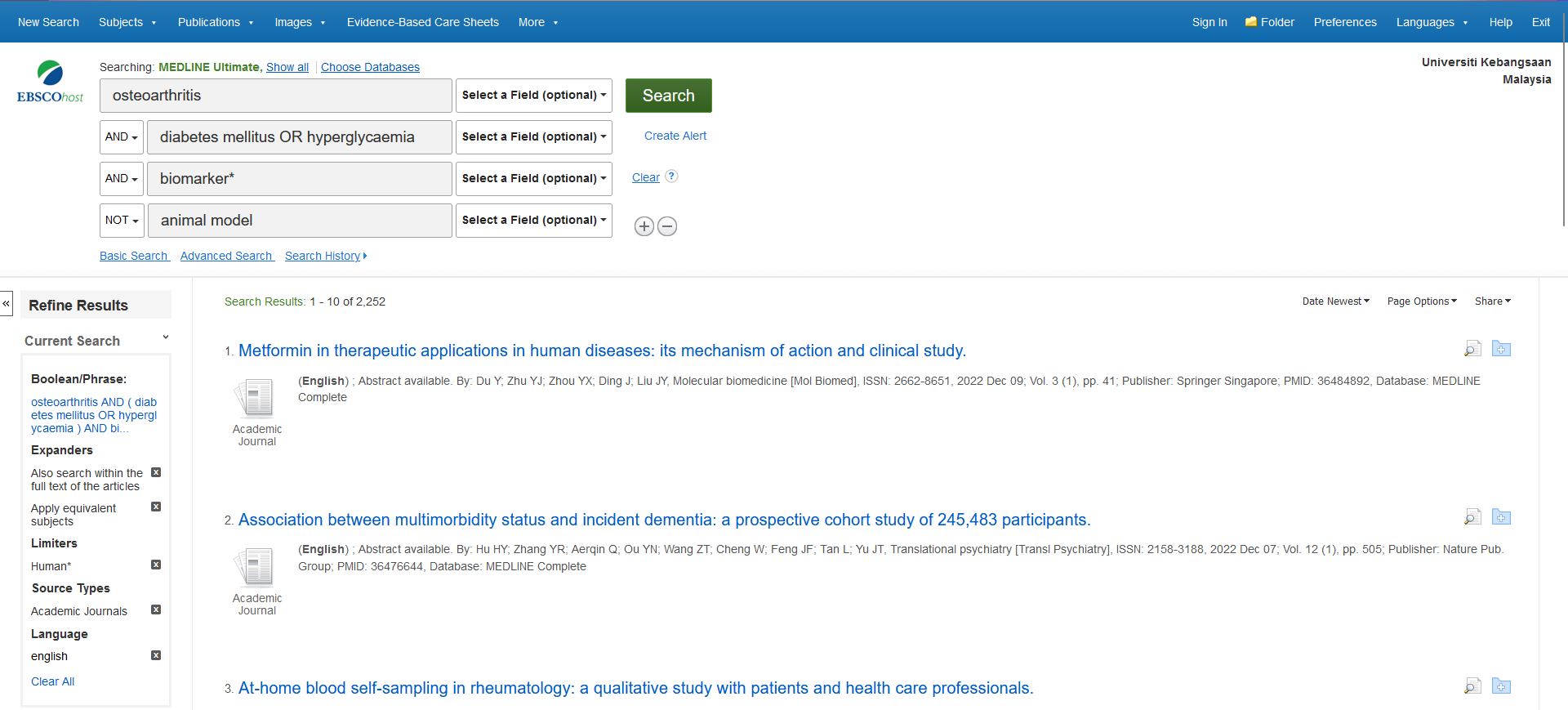


Table S 6 Modified NEWCASTLE - OTTAWA QUALITY ASSESSMENT SCALE Adopted for cross-sectional studies in Biomarkers in Diabetes-Induced-Osteoarthritis: A Scoping Review. Note: A study can be awarded a maximum of one star for each numbered item within the Selection and Outcome categories. A maximum of two stars can be given for Comparability.

| Participant Selection and sampling | | Condition to award a star |
| --- | --- | --- |
| 1 | Representativeness of the study cohort |  |
|  | a) Yes, representative of the average osteoarthritic patients in the community | * |
|  | b) Somewhat representative of the average osteoarthritic patients in the community | * |
|  | c) ) No description of the study cohort |  |
| 2 | Selection of non-exposed cohort |  |
|  | a) Drawn from the same community as the exposed cohort* | * |
|  | b) Drawn from a different source |  |
|  | c) No description of the derivation of the non-exposed cohort |  |
| 3 | Ascertainment of exposure (osteoarthritis and diabetes mellitus) |  |
|  | a) Secure record (eg surgical records) * | * |
|  | b) Structured interview * | * |
|  | c) Written self-report |  |
|  | d) No description |  |
| 4 | Standardised measurement |  |
|  | a) Study uses standardised / validated data acquisition and processing methods * | * |
|  | b) Study did not use standardised / validated data acquisition or/ and processing methods |  |
| Comparability: (Maximum 2 stars: It is possible to select both the responses at this question or none of them) | | Condition to award a star |
| 5 | Comparability on the basis of the design or analysis |  |
|  | a) Study controls for the age, sex or presence of osteoarthritis | * |
|  | b) Study controls for any additional factor (e.g. metabolic syndrome components) | * |
| Outcome | | Condition to award a star |
| 6 | Biomarker identification and annotation |  |
|  | a) Level 1 identification confidence reported (Level 1 identification necessitates that 2 or more orthogonal properties of an authentic chemical standard analysed in the researcher’s laboratory are compared to experimental data acquired in the same laboratory with the same analytical methods) | * |
|  | b) Only putatively annotated / unknown compounds were reported |  |
| 7 | Sample size |  |
|  | a) >=90 * | * |
|  | b) < 90 |  |
| 8 | Statistical test (Maximum 2 stars: It is possible to select one or two of the responses at this question or none of them) |  |
|  | a) Probability level (P value) or confidence interval, and effect size are reported for individual metabolites * | * |
|  | b) If univariate statistics is used, were associations adjusted for multiple testing? * | * |
|  | c) If multivariate statistics is used, has the multivariate model been cross-validated against independent test set?* | * |

Table S 7 Modified NEWCASTLE - OTTAWA QUALITY ASSESSMENT SCALE Adopted for experimental studies in Biomarkers in Diabetes-Induced-Osteoarthritis: A Scoping Review. Note: A study can be awarded a maximum of one star for each numbered item within the Selection and Outcome categories. A maximum of two stars can be given for Comparability.

| Participant Selection and sampling | | Condition to award a star |
| --- | --- | --- |
| 1 | Representativeness of the study population |  |
|  | a) Yes, representative of the average osteoarthritic patients in the community | * |
|  | b) Somewhat representative of the average osteoarthritic patients in the community | * |
|  | b) No description of the study population |  |
| 2 | Test and control group treatment |  |
|  | a) Primary cell cultures were treated equally aside from hyperglycemia stimulation in test group* | * |
|  | b) Test group and control group were not cultured in a controlled way to observe only effect of hyperglycemia stimulation |  |
|  | c) No clear description of the cell culture treatment |  |
| 3 | Ascertainment of exposure (osteoarthritis) |  |
|  | a) Secure record (eg surgical records) * | * |
|  | b) Structured interview * | * |
|  | c) Written self-report |  |
|  | d) No description |  |
| 4 | Standardised measurement |  |
|  | a) Study uses standardised / validated data acquisition and processing methods * | * |
|  | b) Study did not use standardised / validated data acquisition or/ and processing methods |  |
| Comparability: (Maximum 2 stars: It is possible to select both the responses at this question or none of them) | | Condition to award a star |
| 5 | Comparability on the basis of the design or analysis |  |
|  | a) Study controls for the age, sex or presence of osteoarthritis | * |
|  | b) Study controls for any additional factor (e.g. metabolic syndrome components) | * |
| Outcome | | Condition to award a star |
| 6 | Biomarker identification and annotation |  |
|  | a) Level 1 identification confidence reported (Level 1 identification necessitates that 2 or more orthogonal properties of an authentic chemical standard analysed in the researcher’s laboratory are compared to experimental data acquired in the same laboratory with the same analytical methods) | * |
|  | b) Only putatively annotated / unknown compounds were reported |  |
| 7 | Sample size |  |
|  | a) >=10 * | * |
|  | b) < 10 |  |
| 8 | Statistical test (Maximum 2 stars: It is possible to select one or two of the responses at this question or none of them) |  |
|  | a) Probability level (P value) or confidence interval, and effect size are reported for individual metabolites * | * |
|  | b) If univariate statistics is used, were associations adjusted for multiple testing? * | * |
|  | c) If multivariate statistics is used, has the multivariate model been cross-validated against independent test set?* | * |

Table S 8 Risk of bias in individual studies using the modified Newcastle-Ottawa scale. Studies in uncoloured background are cross-sectional studies while studies in blue background are experimental studies.

| **num** | **Author, Year** | **Item*** | | | | | | | | **Total score (max 10)** |
| --- | --- | --- | --- | --- | --- | --- | --- | --- | --- | --- |
|  |  | **1** | **2** | **3** | **4** | **5** | **6** | **7** | **8** |  |
| 1 | (Luo et al., 2022) | * | * | * | * | ** | * |  | * | 8 |
| 2 | (Li et al., 2021) | * | * | * | * | * | * |  | * | 7 |
| 3 | (Scotece et al., 2020) | * | * | * | * | * | * | * | * | 8 |
| 4 | (Silawal et al., 2019) | * | * | * | * |  | * |  | * | 6 |
| 5 | (Vertti, Muiz, Martnez, Galarza, & Astorga, 2019) | * | * | * | * | ** | * | * | ** | 10 |
| 6 | (Eitner et al., 2017) | * | * | * | * | ** | * |  | ** | 9 |
| 7 | (W. Zhang et al., 2017) | * | * | * | * | ** | * |  | * | 8 |
| 8 | (Hamada et al., 2016) | * | * | * | * |  | * | * | * | 7 |
| 9 | (Ribeiro, López de Figueroa, Blanco, Mendes, & Caramés, 2016) | * | * | * | * |  | * | * | * | 7 |
| 10 | (W. D. Zhang et al., 2016) | * | * | * | * | ** | * | * | ** | 10 |
| 11 | (Laiguillon et al., 2015) | * | * | * | * | ** | * | * | * | 9 |
| 12 | (Tsai, Manner, & Li, 2013) | * | * | * | * |  | * |  | * | 6 |
| 13 | (C.-H. Tsai et al., 2013) | * | * | * | * |  | * | * | * | 7 |
| 14 | (Rosa et al., 2011) | * | * | * | * |  | * | * | * | 7 |
| 15 | (Oren, Botolin, Williams, Bucknell, & King, 2011) | * | * | * | * | ** | * | * | ** | 10 |
| 16 | (Rosa et al., 2009) | * | * | * | * |  | * | * | * | 7 |

**Items are available in Table S 6 and 7*

Table S9: Summary table of cross-sectional studies. Studies in grey background are experimental studies while white background are cross-sectional studies.

| **Author, year** | **Country** | **Sample size** | **Study Type** | **Age, years**  **Median (IQR)/ Mean±sd** | **Specimen** | **Detection Method/ Test** | **Statistical analysis** |
| --- | --- | --- | --- | --- | --- | --- | --- |
| (Luo et al., 2022) | China | OA+DM+=5  OA+DM-=5  Control=5 | Cross-sectional study | OA+DM+= 63 (34–80)  OA+DM-= 66 (54–70)  Control= 41 (30-67) | Cartilage | Liquid chromatography coupled with tandem mass spectrometry (LC-MS/MS) | Wilcoxon non-parametric test and  two-sided Fisher’s exact test |
| (Li et al., 2021) | China | OA+DM+=10  OA+DM-=10 | Cross-sectional study | 50-70 | Synovial tissue | Immunohistochemical staining | Mann–Whitney U test |
| (Scotece et al., 2020) | Finland | 100 OA | Cross-sectional study | 72 (14) | Cartilage | Immunoassay | t-test and Spearman’s rank correlation coefficient (r) |
| (Silawal et al., 2019) | Germany | 5 OA | Experimental study | 68.8 ± 20.52 | Human articular chondrocytes and chondrosarcoma cell line | Immunohistochemical staining | Kruskal–Wallis followed by Dunn’s post hoc multiple comparisons or one-way ANOVA |
| (Vertti, Muiz, Martnez, Galarza, & Astorga, 2019) | Mexico | OA+DM+=92  OA+DM-=29 | Cross-sectional study | KOA=62 (42–87)  non-KOA=51.5 (30–76) | Synovial fluid and blood | ELISA | Bivariate analysis with chi-square and Mann–Whitney U tests  Multivariate logistic regression  Kruskal–Wallis |
| (Eitner et al., 2017) | Germany | OA+DM+=23  OA+DM-=47 | Cross-sectional study | 69 ± 14 | Synovial fluid and serum | ELISA | Mann–Whitney U test with Bonferroni, binary logistic regression analysis with dichotomous variables, and multivariate linear regression adjusted using Spearman´s rank correlation coefficient |
| (W. Zhang et al., 2017) | Canada | OA+DM+= 46  OA+DM-=38 | Cross-sectional study | OA+DM+= 68±6.6  OA+DM-= 62.7±8.8 | Synovial fluid and plasma | Mass spectrometry by flow injection analysis (MS-FIA)  Liquid chromatography coupled with tandem mass spectrometry (LC-MS/MS) | t-test and Spearman correlation coefficient adjusted by linear regression modelling |
| (Hamada et al., 2016) | USA | OA+DM+=7  OA+DM-=6  (Synovial tissue)  OA+DM-=4  (FLS culture) | Experimental study | OA+DM+= 63.3±5.8  OA+DM-=62.3±7.7  (Synovial tissue)  56–87(FLS culture) | Synovial tissue and fibroblast-like-synoviocytes culture | Immunohistochemistry, immunoprecipitation, western blot analysis and qRT-PCR | Two ways or one way ANOVA followed by Sidak’s or Dunnett’s post hoc test, respectively |
| (Ribeiro, López de Figueroa, Blanco, Mendes, & Caramés, 2016) | Spain | OA+DM+=4  OA+DM-=4  Control=2 | Experimental study | OA+DM+=70.75±10.17  OA+DM-=78.75±11.52  Control=54±1.41 | Primary human chondrocytes | Western blot | Student's unpaired t-test or ANOVA in conjunction with Tukey's multiple comparison |
| (W. D. Zhang et al., 2016) | Canada | OA+DM+=29  OA+DM-=43  DM=25  Control=46 | Cross-sectional study | OA+DM+=63±5.7  OA+DM-=62±4.8  DM=60±6.6  Control=61±6.5 | Synovial fluid and plasma | UPLC-MS coupled with assay kit | Orthogonal projection to latent structure-discriminant analysis, Kruskal–Wallis test further retested with S-plot, and linear regression with Bonferroni |
| (Laiguillon et al., 2015) | France | OA+DM+ =5  OA+DM- =5 | Experimental study | OA+DM+=62±7.8  OA+DM-=69.6±8.6 | Homogenous isolated cartilage samples | ELISA | Wilcoxon paired test, Mann-Whitney test and Chi-square test |
| (Tsai, Manner, & Li, 2013) | USA | 3 OA | Experimental study | 25-50 | Human bone marrow derived mesenchymal stem cell | Western blot | T-test |
| (C.-H. Tsai et al., 2013) | China | 35 OA | Experimental study | - | Human synovial fibroblast culture | qPCR and ELISA | Mann-Whitney U test for non-Gaussian parameters |
| (Rosa et al., 2011) | Portugal | 11 OA  7 non-OA | Experimental study | OA= 50–71  Non-OA= 18–40 | Cartilage chondrocytes culture | qPCR | Paired two-tailed Student t-test |
| (Oren, Botolin, Williams, Bucknell, & King, 2011) | USA | OA+DM+=10  OA+DM-=10 | Cross-sectional study | 45-80 | Synovial fluid, serum, bone and cartilage | ELISA and high-performance liquid chromatography | Unpaired Student's t test and two ways ANOVA followed Bonferroni post test |
| (Rosa et al., 2009) | Portugal | Non-OA=15  OA= 18 | Experimental study | Non-KOA=28-35  KOA= 52-77 | Cartilage chondrocytes culture | qPCR | Unpaired two-tailed t-test or Mann-Whitney rank sum test, Pearson product moment correlation test, and multiple linear regression |

Table S10: Reported biomarkers associated with DM-OA and their magnitude changes as compared to OA biomarkers.

| **Class/pathway** | **Metabolite** | **Ref. of study reporting increase** | **Ref. of study reporting decrease** |
| --- | --- | --- | --- |
| Advanced glycation end-products | Methylglyoxal (MG) | 7 |  |
| Advanced glycation end-products | Free methylglyoxal-derived hydroimidazolone-1 (MG-H1) | 7 |  |
| Advanced glycation end-products | Pentosidine | 15 |  |
| Cartilage turnover factor | Aggrecan |  | 12 |
| Cartilage turnover factor | Collagen type I |  | 4 |
| Cartilage turnover factor | Proteoglycan |  | 4 |
| Cartilage turnover factor | Synovial Fluid Cartilage Oligomeric Matrix Protein (SF COMP) | 5 |  |
| Cartilage turnover factor | Collagen type II | 4, 14 | 12 |
| Cell homeostasis regulators | SRY-Box Transcription Factor 9 (SOX9) |  | 4, 12 |
| Cell homeostasis regulators | Akt |  | 8 |
| Cell homeostasis regulators | Microtubule-associated protein 1A/1B-light chain 3 (LC3) |  | 9 |
| Cell homeostasis regulators | Phosphorylated ribosomal S6 (p-rpS6) | 9 |  |
| Cell homeostasis regulators | Protein Kinase C (PKC) | 12 |  |
| Cell homeostasis regulators | Type II TGF-b receptor (TGFbRII) | 12 |  |
| Cell homeostasis regulators | vascular endothelial growth factor (VEGF) | 13 |  |
| Cell homeostasis regulators | Glucose regulated protein 78 (GRP78) | 2 |  |
| Cell homeostasis regulators | Activating transcription factor 6 (ATF6) | 2 |  |
| Cell homeostasis regulators | Hypoxia-inducible factor-1α (HIF-1α) | 2 |  |
| Cell homeostasis regulators | C8 alpha chain N437 (C8A) | 1 |  |
| Cell homeostasis regulators | Cluster of differentiation 47 (CD47) |  | 1 |
| Cell homeostasis regulators | Angiopoietin-like protein 2 (ANGPTL2) |  | 1 |
| Cell homeostasis regulators | Basigin (BSG) |  | 1 |
| Cell homeostasis regulators | Biglycan (BGN) |  | 1 |
| Cell homeostasis regulators | Cathepsin D (CTSD) |  | 1 |
| Cell homeostasis regulators | Junctional cadherin 5-associated protein (JCAD) |  | 1 |
| Cell homeostasis regulators | Fibulin-7 (FBLN7) |  | 1 |
| Cell homeostasis regulators | Tenascin 1018 and 184 |  | 1 |
| Cell homeostasis regulators | Thrombospondin 3 (THBS3) |  | 1 |
| Cell homeostasis regulators | Immunoglobulin Heavy Constant Mu (IGHM) |  | 1 |
| Cell homeostasis regulators | Asporin (ASPN) |  | 1 |
| Cell homeostasis regulators | Secreted protein acidic and rich in cysteine/osteonectin (SPARC) |  | 1 |
| Cell homeostasis regulators | Fibronectin 1 (FN1) |  | 1 |
| Cell homeostasis regulators | Collagen type I alpha 1 chain (COL1A1) |  | 1 |
| Cell homeostasis regulators | Collagen type VI alpha 1 chain (COL6A2) |  | 1 |
| Diabetes mellitus specific biomarkers | Retinol binding protein 4 (RBP4) | 3 |  |
| Diabetes mellitus specific biomarkers | Fasting plasma glucose | 5 |  |
| Diabetes mellitus specific biomarkers | Haemoglobin A1c (HbA1c) | 5, 15 |  |
| Diabetes mellitus specific biomarkers | Advanced glycation end products (AGEs) | 2 |  |
| Diabetes mellitus specific biomarkers | Insulin receptor (IR) |  | 8 |
| Diabetes mellitus specific biomarkers | glucose transporter 1 (GLUT1) | 2 | 16 |
| Inflammatory mediators | Nuclear factor kappa B (NF-κB p65) | 2 |  |
| Inflammatory mediators | ADAM Metallopeptidase With Thrombospondin Type 1 Motif 5 (ADAMTS5) | 2 |  |
| Inflammatory mediators | Interleukin-6 (IL-6) | 2, 6, 11 |  |
| Inflammatory mediators | Tumour Necrosis Factor alpha (TNF-α) | 2, 8 |  |
| Inflammatory mediators | ADAM Metallopeptidase With Thrombospondin Type 1 Motif 4 (ADAMTS4) | 8 |  |
| Inflammatory mediators | Reactive oxygen species (ROS) | 16 |  |
| Inflammatory mediators | prostaglandin E2 (PGE2) | 11 |  |
| Phosphatidylcholines | PC ae C34:3 |  | 10 |
| Phosphatidylcholines | PC ae C36:3 |  | 10 |
| Protease | Tissue inhibitor of metalloproteinases 1 (TIMP-1) | 14 |  |
| Protease | Tissue inhibitor of metalloproteinases 2 (TIMP-2) |  | 14 |
| Protease | Metalloproteinase 1 (MMP1) | 8, 14 |  |
| Protease | Metalloproteinase 13 (MMP13) | 2, 8, 14 |  |

**Abbreviation List**

| ACR | American College of Rheumatology |
| --- | --- |
| ADAMTS4 | A disintegrin and metalloproteinase with thrombospondin motifs 4 |
| ADAMTS5 | A disintegrin and metalloproteinase with thrombospondin motifs 5 |
| AGEs | Advanced glycation end products |
| Akt | Serine threonine kinase |
| AMPK | AMP-activated protein kinase |
| ANGPTL2 | Angiopoietin-like protein 2 |
| ASPN | Asporin |
| ATF6 | Activating transcription factor 6 |
| BGN | Biglycan |
| BIPED | Burden of Disease, Investigative, Prognostic, Efficacy of Intervention and Diagnostic Biomarkers |
| BSG | Basigin |
| C8A | C8 alpha chain N437 |
| CD47 | Cluster of differentiation 47 |
| CHI3L1 | Chitinase 3-like 1 |
| COL1A1 | Collagen type I alpha 1 chain |
| COL6A2 | Collagen type VI alpha 1 chain |
| CTSD | Cathepsin D |
| DALYs | Disability adjusted life years |
| DM | Diabetes mellitus type 2 |
| DM-OA | Diabetes mellitus-induced osteoarthritis |
| ELISA | Enzyme-linked immunosorbent assay |
| FBLN7 | Fibulin-7 |
| FN1 | Fibronectin 1 |
| GLUT-1 | Glucose transporter 1 |
| GRP78 | Glucose regulated protein 78 |
| hAC | Human articular chondrocytes |
| HbA1c | Glycated haemoglobin |
| HIF-1α | Hypoxia-inducible factor-1α |
| IGHM | Immunoglobulin Heavy Constant Mu |
| IL-10 | Interleukin-10 |
| IL-1β | Interleukin-1β |
| IL-6 | Interleukin-6 |
| IR | Insulin receptor |
| JCAD | Junctional cadherin 5-associated protein |
| KL grading | Kellgren and Lawrence system grading |
| LC3 | Microtubule-associated protein 1A/1B-light chain |
| LC-MS/MS | Liquid chromatography coupled-tandem mass spectrometry |
| MAPK | Mitogen-activated protein kinase |
| MG | Methylglyoxal |
| MG-H1 | Free methylglyoxal-derived hydroimidazolone-1 |
| MMP-1 | Matrix metalloproteinase-1 |
| MMP-3 | Matrix metalloproteinase-3 |
| MMP-13 | Matrix metalloproteinase-13 |
| mRNA | Messenger ribonucleic acid |
| mTORC1 | Mammalian target of rapamycin complex 1 |
| NADPH | Nicotinamide adenine dinucleotide phosphate |
| NF-κB | Nuclear factor kappa-light-chain-enhancer of activated B cells |
| NF-κB p65 | Rela of nuclear factor kappa-light-chain-enhancer of activated B cells |
| OA | Osteoarthritis |
| PKC | Protein kinase C |
| p-rpS6 | Phosphorylated ribosomal S6 |
| qRT-PCR | Quantitative real time polymerase chain reaction |
| RAGE | Receptor for advanced glycation end products |
| RBP4 | Retinol binding protein 4 |
| ROS | Reactive oxygen species |
| SF COMP | Synovial fluid cartilage oligomeric matrix protein |
| Smad3 | SMAD family member 3 |
| SPARC | Secreted protein acidic and rich in cysteine/osteonectin |
| SOX9 | Sry-box transcription factor 9 |
| TGFbRII | Type II TGF-b receptor |
| TGF-β | Transforming growth factor-β |
| THBS3 | Thrombospondin 3 |
| TIMP-1 | Tissue inhibitor of metalloproteinases-1 |
| TIMP-2 | Tissue inhibitor of metalloproteinases-2 |
| TNC | Tenascin C |
| TNF-α | Tumor necrosis factor-α |
| VEGF | Vascular endothelial growth factor |
| YLDs | Years Lived with Disability |
